# Flow-based In Vivo Method to Enumerate Translating Ribosomes and Translation Elongation Rate

**DOI:** 10.21769/BioProtoc.5165

**Published:** 2025-01-20

**Authors:** Mina O. Seedhom, Devin Dersh, Jonathan W. Yewdell

**Affiliations:** 1 Diabetes Center of Excellence, Department of Molecular Medicine, University of Massachusetts Medical School, Worcester, MA, USA; 2 Department of Biochemistry and Biophysics, Perelman School of Medicine, University of Pennsylvania, Philadelphia, PA, USA; 3 National Institute of Allergy and Infectious Diseases, Bethesda, MD, USA

**Keywords:** Protein synthesis, Puromycin, Ribosome, Nascent chain, **R**ibo**P**uro**M**ycylation (RPM), Ribosome transit analysis (RTA)

## Abstract

Protein synthesis is by far the most energetically costly cellular process in rapidly dividing cells. Quantifying translating ribosomes in individual cells and their average mRNA transit rate is arduous. Quantitating assembled ribosomes in individual cells requires electron microscopy and does not indicate ribosome translation status. Measurement of average transit rates entails in vitro pulse-chase radiolabeling of isolated cells or ribosome profiling after ribosome runoff, which is expensive and extremely demanding technically. Here, we detail protocols based on ribosome-mediated nascent chain puromycylation, harringtonine to stall initiating ribosomes while allowing ribosome elongation to continue normally, and cycloheximide to freeze translating ribosomes in place. Each compound is delivered intravenously to mice in the appropriate order, and after ex vivo cell fixation and permeabilization, translating ribosome numbers and transit rates are measured by flow cytometry using a directly conjugated puromycin-specific antibody.

Key features

• Measure relative numbers of translating ribosomes in mixed single-cell preparations.

• Quantitate relative in vivo ribosome transit rates in mixed single-cell preparations.

• Detect ribosome stalling in vivo.

## Graphical overview


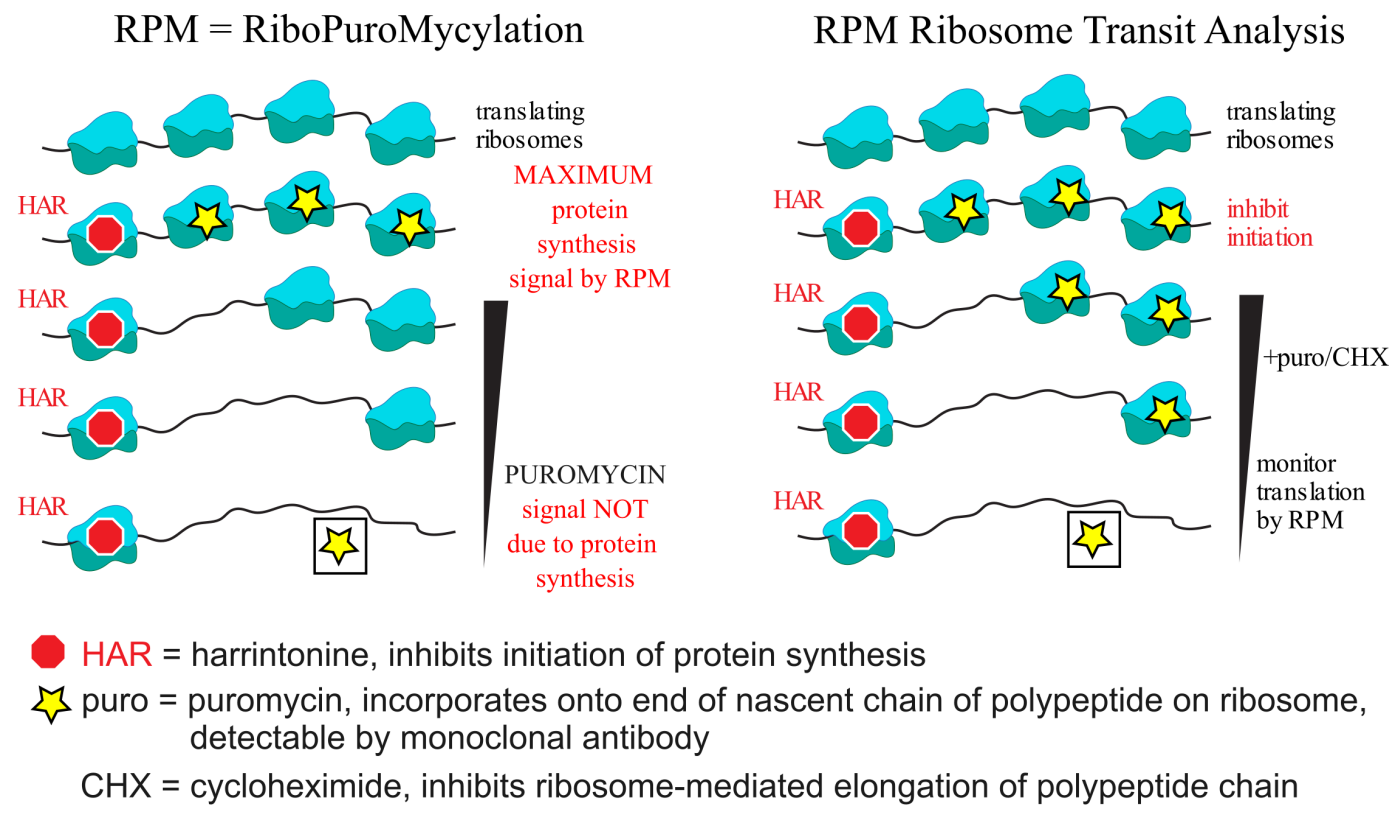


## Background

Proteins are the most abundant macromolecule by mass and copy number in nearly all cell types in varied proliferation states [1,2]. Understanding proteostasis, the net outcome of ribosomal synthesis, folding, secretion/release, and degradation, requires an accurate accounting of ribosome numbers and translation rates.

The protocol we describe here is based on puromycin (PMY), a remarkable aminonucleoside antibiotic that interferes with protein synthesis in all forms of life. By mimicking an aminoacylated tyrosyl tRNA, PMY is incorporated by ribosome catalysis into nascent chains, where it terminates elongation as it lacks a COOH terminus that can form a peptide bond [3,4]. This reaction, termed puromycylation, occurs spontaneously, even at 0 °C. It has been utilized (in conjunction with various protein translation modulators) by our laboratory to uncover evidence of nuclear translation [5], antigen-independent cytokine-induced division of memory-like T cells in the bone marrow of mice after virus infection [6], and stalled ribosomes in neurons and immune cells [7,8], along with many discoveries by other labs [9].

We recently reported protocols that measure the numbers of translating ribosomes and ribosome transit rates in mice based on intravenous (IV) delivery of protein synthesis modulators and flow cytometry–based detection of puromycylated nascent chains using a fluorescently labeled anti-puromycin antibody (Ab) [8]. Herein, we describe these protocols in detail.

## Materials and reagents

**Biological materials**

1. *C57BL/6*J mice, male or female (strain number: 00664)
2. BD Pharmingen^TM^ purified rat anti-mouse CD16/CD32 (mouse BD Fc Block™) (BD Bioscience, catalog number: 553141)

**Reagents**

1. Puromycin dihydrochloride from *Streptomyces alboniger* (PMY) (Sigma-Aldrich, catalog number: P8833-100MG)
2. Cycloheximide (CHX) (EMD Millipore, catalog number: 239764, 100 mg)
3. Methanol (certified ACS) (Fisher Chemical, catalog number: A412-4)
4. Harringtonine (HAR) (Santa Cruz Biotech, catalog number: sc-204771A)
5. RPMI 1640 medium, GlutaMAX^TM^ supplement (Thermo Fisher Scientific, catalog number: 61870036)
6. DPBS, no calcium, no magnesium (Thermo Fisher Scientific, catalog number: 14190144)
7. Anti-puromycin Ab, produced from in-house-generated hybridoma (Developmental Studies Hybridoma Bank, catalog number: PMY-2A4)
8. ACK lysing buffer (Lonza, catalog number: A1049201)
9. Trypan Blue solution, 0.4% (Thermo Fisher Scientific, catalog number: 15250061)
10. Fujifilm Wako chemicals USA digitonin 1 g (Fisher Scientific, catalog number: NC0141730)
11. Paraformaldehyde, 16% w/v aq. soln., methanol free (Thermo Fisher Scientific, catalog number: 043368.9M)
12. Fluorescent Protein Labeling kits (Thermo Fisher Scientific, catalog number: A10235)
13. Ethidium monoazide bromide (EMA) (Thermo Fisher Scientific, catalog number: E1374)
14. Bovine serum albumin (BSA) (Millipore Sigma, catalog number: A7888-10G)
15. Sodium azide (Millipore Sigma, catalog number: 71290)
16. Ethyl alcohol, pure (Millipore Sigma, catalog number: 459836-100ML)
17. Fetal bovine serum (FBS) (Atlanta Biologicals, catalog number: S10350)

**Solutions**

1. RPMI with 7.5% fetal bovine serum (see Recipes)
2. Puromycin (PMY), cycloheximide (CHX), and harringtonine (HAR) solution for IV injections (1 mL) (see Recipes)
3. Stock CHX solution (see Recipes)
4. Stock HAR solution (see Recipes)
5. Stock PMY solution (see Recipes)
6. HAR/PMY/CHX solution for intravenous injection (see Recipes)
7. HAR solution for intravenous injection (see Recipes)
8. Fluorescence-activated cell sorting (FACS) buffer (see Recipes)
9. Stock EMA solution (see Recipes)
10. Stock digitonin solution (see Recipes)
11. Fixation and permeabilization buffer (see Recipes)

**Recipes**

1. **RPMI 1640 with 7.5% FBS**
  500 mL of RPMI 1640 with GlutaMAX
  37.5 mL of FBS
  Store at 4 °C.
2. **Puromycin (PMY), cycloheximide (CHX), and harringtonine (HAR) solution for IV injections (1 mL)**
  1 mg of PMY, 100 μg of HAR, and 0.34 mg of CHX per mouse; 100 μL per mouse; make fresh from stocks.
  20 μL of HAR stock solution
  200 μL of PMY stock solution
  170 μL of CHX stock solution
  610 μL of PBS
  Warm to 37 °C before use to allow PMY to fully solubilize, then keep at room temperature for up to 5 h.
3. **Harringtonine (HAR) solution for IV infections (1 mL)**
  100 μg of HAR per mouse, 100 μL per mouse, make fresh from stocks.
  20 μL of HAR stock solution
  980 μL of PBS
  Once made, use immediately. Can remain at room temperature for ~5 h.
4. **CHX stock solution in 50% ethanol (20 mg/mL)**
  100 mg of CHX
  5 mL of 50% ethanol, 50% water
  Aliquot and store at -20 °C.
5. **PMY stock solution in PBS (50 mg/mL)**
  100 mg of puromycin (PMY)
  2 mL of PBS
  Warm to 37 °C to solubilize, aliquot (we usually use 1 mL), and store at -20 °C.
6. **Harringtonine (HAR) stock solution in methanol (50 mg/mL)**
  10 mg of HAR
  200 μL of methanol
  Aliquot and store at -20 °C.
7. **1% digitonin stock solution in DMSO**
  1 g of digitonin
  100 mL of DMSO
  Aliquot and store at -20 °C.
8. **Fixation/permeabilization buffer (1% paraformaldehyde, 0.0075% digitonin)**
  9.3 mL of PBS
  625 μL of 16% paraformaldehyde
  75 μL of 1% digitonin
  Make fresh and keep on ice.
9. **FACS buffer (0.2% sodium azide, 1 mg/mL bovine serum albumin)**
  1 g of sodium azide
  500 mg of BSA
  500 mL of PBS
  Store at 4 °C.
10. **EMA stock solution (5 mg/mL)**
  5 mg of EMA
  1 mL of DMSO
  Aliquot and store at -20 °C.
11. **Ethidium monoazide solution for staining (10 μg/mL)**
  20 μL of EMA stock solution
  10 mL of PBS
  Make fresh and keep on ice.

**Laboratory supplies**

1. Falcon^TM^ 15 mL conical polypropylene centrifuge tubes (Fisher Scientific, catalog number: 14-959-49B)
2. Corning^®^ Primaria^ TM^ 60 mm × 15 mm standard cell culture dish (Corning, catalog number: 353802)
3. Microscope slides, glass, 25 × 75 mm, 90° ground edges, frosted, 1 end, both sides (Globe Scientific, catalog number: 1308)
4. Falcon^®^ 70 μm cell strainer, white, sterile, individually packaged, 50 per case (Fisher Scientific, catalog number: 22-363-548)
5. Cellometer SD025 slides, box of 75 slides (Revvity, catalog number: CHT4-SD025-002)
6. Corning^®^ 96-well clear round bottom TC-treated microplate (Corning, product number: 3799)
7. BD Eclipse^TM^ needle 27 G × 1/2 in. with detachable 1 mL BD Luer-Lok^TM^ syringe (BD Biosciences, GTIN number: 00382903057894)

## Equipment

1. Cellometer X2 fluorescent viability counter (Nexcelom, catalog number: CMT-X2-S150)
2. Tailveiner restrainer for mice (Braintree Scientific, Inc., SKU: TV-150)
3. Infrared heating and drying lamp, table model (Walter Stern, VWR, Avantor, catalog number: 36547-009)
4. Fisherbrand^TM^ accuSpin Max 1.6 L benchtop centrifuge (Fisher Scientific, catalog number: 75-883-78)
5. LSR Fortessa X-20 (BD Bioscience, catalog number: 50165)

## Software and datasets

1. Microsoft Excel for Mac, version 16.85
2. GraphPad Prism 10 for MacOS
3. FlowJo 10.10.0 for Mac OS X

## Procedure

**A. Intravenous injections of reagents necessary for in vivo RiboPuroMycylation (RPM**)

*Note 1: All in vivo procedures require approval by your institutional Animal Care and Use Committee (ACUC).*

*Note 2: The in vivo RPM procedure has been successfully performed on lymph nodes, spleens, and thymi from mice, although a similar procedure has been performed in vitro on other organs such as fetal liver [10].*

*Note 3: For in vivo RPM and RTA procedures, we have used female and male mice aged 1.5–18 months. Mice were housed on a 12 h light/dark cycle with ad libitum access to normal mouse chow and water. The procedure was developed using C57BL/6J (B6) mice, and we have performed it using other mice on the B6 and FVB/N background. We expect this protocol will work similarly in other mouse strains and animal models, with adjustments to drug dosage.*

1. For the in vivo RPM assay, prepare a solution of 10 mg/mL PMY, 1 mg/mL HAR, and 3.4 mg/mL CHX in PBS and warm in a water bath to 37 °C to allow PMY to completely solubilize (see Recipes). Prepare approximately 20% extra of this solution, as fluid loss during injections will occur.
2. Prepare a solution of 1 mg/mL of HAR in PBS (see Recipes). Prepare approximately 20% extra of this solution, as fluid loss during injections will occur.
3. Warm mice for 5–10 min using an ACUC-approved heat lamp to dilate tail veins. Mice are considered sufficiently warm for injections when their activity within the cage is increased.
4. Restrain warmed mice in a mouse holder and intravenously inject the tail vein, using a 27G × ½ needle attached to a 1 mL syringe, with 100 μL of the HAR/CHX/PMY solution, waiting 5 min for the maximum signal. To determine the background RPM signal not related to active protein synthesis, inject mice with 100 μL of the HAR solution, wait 15 min for ribosomes to finish translating mRNAs, and then inject 100 μL of the HAR/CHX/PMY solution and wait 5 min. A third control is necessary, injecting vehicle without PMY to control for non-specific staining of the anti-PMY mAb.
5. After the indicated times, sacrifice mice by cervical dislocation and harvest organs of interest into 15 mL conical centrifuge tubes containing 3 mL of RPMI supplemented with 7.5% FBS on ice.
  **Figure 1.**
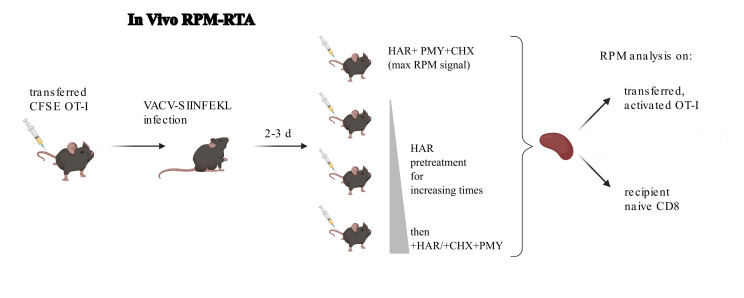
Depiction of the in vivo RiboPuroMycylation (RPM)-ribosome transit assay (RTA). In this example, CFSE-labeled OT-1 T cells (CFSE tracks cell division) are adoptively transferred into congenic mice (to allow tracking of donor cells in recipient mice), followed by infection with vaccinia virus expressing the SIINFEKL peptide (Kb-SIINFEKL activates OT-1 T cells). RPM-RTA is performed by intravenous injection of harringtonine (HAR) for different amounts of time followed by HAR, cycloheximide (to prevent leakiness from HAR inhibition alone), and puromycin. Spleens are harvested for RPM analysis on both endogenous and transferred T cells. Schematic designed with BioRender.

**B. Intravenous injections of reagents necessary for in vivo RPM ribosome transit analysis (RTA)**

*Note: The timing of intravenous injections is critical. It is strongly suggested that this procedure be done by a two-person team: one person using a timer to record the time of injections and the other to perform the injections.*

1. For in vivo RPM RTA (depiction of an experimental setup in Figure 1), prepare a solution of 10 mg/mL PMY, 1 mg/mL HAR, and 3.4 mg/mL CHX and warm in a water bath to 37 °C to allow PMY to completely solubilize (see Recipes). Prepare approximately 20% extra of this solution, as fluid loss during injections will occur.
2. Prepare a solution of 1 mg/mL HAR (see Recipes). Prepare approximately 20% extra of this solution, as fluid loss during injections will occur.
3. Warm mice for 5–10 min using an ACUC-approved heat lamp to dilate tail veins. Mice are considered sufficiently warm for injections when their activity within the cage is increased.
4. Restrain warmed mice in a mouse holder, intravenously inject the tail vein using a 27G × ½ needle attached to a 1 mL syringe with 100 μL of the HAR/CHX/PMY solution, and wait 5 min for maximum signal. To determine ribosome transit times, inject 100 μL of HAR and wait for 30 s, 1 min, 2 min, 4 min, or 10 min before injecting with 100 μL of the HAR/CHX/PMY solution. As above, a no-PMY control is needed.
5. After the indicated times, sacrifice mice by cervical dislocation and harvest organs of interest into 15 mL conical centrifuge tubes containing 3 mL of RPMI supplemented with 7.5% FBS on ice.

**C. Preparation of single-cell samples for flow cytometry**

*Note 1: All steps below are performed at 4 °C with ice-cold solutions.*

*Note 2: We have stained anywhere from 1 to 6 million cells per well in a 96-well plate, with adjustments to antibody concentrations for surface and intracellular antigens.*

1. Crush organs (we have used spleens, thymi, or lymph nodes) in 3 mL of RPMI between two frosted microscope slides in a 60 mm × 15 mm standard cell culture dish. Filter resultant single-cell suspensions through a 70 μm mesh screen back into a standard polyethylene 15 mL centrifuge tube.
2. Lyse red blood cells present in tissue samples by adding 6 mL of ACK lysing buffer directly to the single cell suspensions and mix by quickly rotating the tubes.
3. Centrifuge filtered single-cell suspensions at 350× *g* for 4 min at 4 °C and pour off supernatants. Disrupt cell pellets by tapping the bottom of the 15 mL conical centrifuge tubes quickly 3–4 times. Resuspend cells in 3 mL of cold RPMI supplemented with 7.5% FBS. Centrifuge at 350× *g* for 4 min at 4 °C, pour off supernatants, disrupt cell pellet, and add 3 mL of cold RPMI supplemented with 7.5% FBS. Refilter cell suspensions again through a 70 μm mesh screen.
4. Count resuspended cells with a Nexcelom Cellometer Vision using Trypan Blue (1:1 dilution) for live/dead cell discrimination as per the manufacturer’s instructions. After counting, centrifuge the cell suspensions in 15 mL conical centrifuge tubes at 350× *g* for 4 min at 4 °C, pour off supernatants, disrupt cell pellets, and resuspend cells in cold RPMI supplemented with 7.5% FBS at 10 million cells per milliliter. Add 200 μL (1–6 million cells per well can be plated; here, 2 million cells per well are plated) of the cell suspensions from each sample to a well of a 96-well polystyrene U-bottom plate.

**D. Surface and intracellular stains for flow cytometry analysis**

*Note 1: Stains for flow cytometry are all performed on ice.*

*Note 2: Anti-puromycin Ab should be conjugated with the required fluorochrome ahead of time with a Life Technologies Protein Labeling kit as per the manufacturer’s instructions.*

*Note 3: The fluorescence signal from the fluorochrome-conjugated anti-puromycin Ab will dim over time. It is recommended that you only use the conjugated anti-PMY Ab for six months.*

1. For single-color controls (SCC) and fluorescence-minus-one (FMO) controls, prepare a mixture of equal numbers of cells from each sample for each organ type isolated (all splenocytes in one mixture, all cells from thymi in a separate mixture). This mixture is made for appropriate flow cytometer setup and downstream analysis. New SCCs and FMOs should be prepared for each experiment. Settings on the flow cytometer may be reused, depending on the experience of the experimenter.
2. Mix Abs at proper dilutions in FACS buffer for SCCs, FMOs, and the full Ab mix to allow for 100 μL per sample in the 96-well plate. Centrifuge cells in the 96-well plate at 350× *g* for 4 min at 4 °C. Before dumping the supernatant [with one (!) quick smooth wrist flick into a biohazard bucket], check that solid cell pellets have formed at the bottom of the 96-well plate.
3. Resuspend cells in 200 μL of PBS using a 12-well multichannel pipette. Centrifuge the plate as above. Dump the supernatant and resuspend the appropriate FMOs, SCCs, and samples in 100 μL of a 10 μg/mL solution of EMA in PBS for flow-based live/dead cell discrimination (see Recipes). Place the plate in the dark on ice for 10 min. Next, expose the uncovered plate close to a fluorescent light with the lid off for an additional 10 min on ice. While exposure to a commercial fluorescent light is suitable for crosslinking EMA to DNA, a wavelength of 465–475 nm has been described to work in the PMA-Lite^TM^ 2.0 LED Photolysis Device (Biotium, catalog number: E90006). Add 100 μL of PBS to each well, pipette up and down to mix, and centrifuge as above. After washing with 200 μL of PBS twice, perform surface staining.
4. Resuspend cells in 100 μL of FACS buffer with the 2.42G monoclonal antibody (Ab) to block Fc receptors (dilution as per manufacturer’s instructions) on ice for 10 min. Add 100 μL of the appropriate single Abs for SCCs, Ab mixes for FMOs, or full Ab mix for full stains, to cell surface antigens at appropriate dilutions for 30 min at 4 °C (a common Ab panel we use and the associated dilutions are listed in Table 1). Centrifuge as above and wash twice in 200 μL of PBS.
  **Table 1.** Example flow cytometry panel

Antibody and fluorochrome	Manufacturer	Catalog number	Dilution
BD Horizon BV786 hamster anti-mouse CD3e (clone 145-2-C11)	BD Bioscience	564379	0.75 μL per test
BD Horizon BV510 rat anti-mouse CD4 (clone RM4-5)	BD Bioscience	563106	0.75 μL per test
BD Horizon PE-CF594 rat anti-mouse CD8a (clone 53-6.7)	BD Bioscience	562283	0.75 μL per test
BD Pharmingen PE mouse anti-mouse Vβ 5.1, 5.2 T-cell receptor (clone MR9-4)	BD Bioscience	562086	0.75 μL per test
eBioscience PE-Cyanine7 rat anti-mouse CD19 (clone eBio1D3 (1D3)	Invitrogen	25-0193-82	0.75 μL per test
eBioscience APC mouse anti-mouse CD45.1 (clone A20)	Invitrogen	17-0453-82	0.75 μL per test
eBioscience mouse anti-mouse Super Bright 780 CD45.2 monoclonal Ab (clone 104)	Invitrogen	78-0454-82	0.75 μL per test
5. Next, simultaneously fix and permeabilize cells in 100 μL of freshly made fixation/permeabilization buffer (fix/perm) (1% PFA, 0.0075% digitonin in PBS, see Recipes) for 20 min at 4 °C. Add 100 μL of PBS, centrifuge as above, and wash twice with PBS.
6. Stain for PMY after the fix/perm step using a 1:100 dilution of the conjugated anti-PMY Ab for 1 h (it could be overnight if more convenient). Add 100 μL of PBS, pipette up and down 6–8 times, and centrifuge the plate as above. Wash twice with 200 μL of PBS, resuspend cell pellets in FACS buffer, and analyze samples (samples should remain on ice during the run) with a flow cytometer. Samples may be stored in a dark refrigerator for up to a week prior to running on a flow cytometer. We use a BD LSRII or BD LSR Fortessa X-20 to run samples, but any flow cytometer with the appropriate lasers and filter sets may be used.

## Data analysis

1. Analyze resulting data using FlowJo, Microsoft Excel, and GraphPad Prism software. For our analysis, we gated OT-1 CD8^+^ T cells by gating on singlets by FSCa and FSCw, lymphocytes by SSCa and FSCa, live cells by EMA-, T cells by CD3^+^CD19-, CD8^+^ T cells by CD8^+^CD4-, adoptively transferred cells by CD45.1^+^CD4-, and OT1 T cells by Vb5^+^CD45.2-, as shown in Figure 2a. For RPM values, we took the population of interest, in this case adoptively transferred OT-1 CD8^+ ^T cells, and determined the mean fluorescence intensity (MFI) of PMY staining in mice treated with different inhibitors of either the CFSE^+^ population in uninfected mice or the CFSE- population in day 3 VACV-SIINFEKL-infected mice. We chose an OT1 T-cell experiment with CFSE labeling (CFSE tracks cell division, CFSE hi are undivided, CFSE low are divided) to distinguish the difference in protein synthesis in actively dividing vs. resting (non-dividing) T cells.
2. For the in vivo RPM assay, subtract the no-PMY MFI in your population of interest (OT1 T cells here) from the HAR/CHX/PMY-treated PMY MFI (maximum signal). Next, subtract the no-PMY PMY MFI signal from the 15-min HAR-treated and then the HAR/CHX/PMY PMY signal.
*Note: Typically, only two no-PMY-treated mice are required for each manipulation, and the average of the RPM MFIs of the no-PMY-treated mice are taken, as the MFI for each no-PMY mouse should be very similar.*
3. Next, subtract the no-PMY-subtracted 15-min HAR-treated and then HAR/CHX/PMY-treated PMY signal from the no-PMY-subtracted HAR/CHX/PMY PMY signal. This will be the relative number of ribosomes in the cell population analyzed (RPM value). If the 15-min HAR-treated and then HAR/CHX/PMY-treated runoff RPM signal is consistently higher than the no-PMY RPM signal, this could be an example of stalled translation, as we have seen in cultured un-activated lymphocytes in vitro [8] and has also been described in neurons [7].
4. For the in vivo RTA assays, the initial analysis is the same, but as the analysis is for different times after runoff, the resultant values are plotted in GraphPad Prism against the time of each individual HAR treatment, and a one-phase decay analysis is performed on the resultant graph. An example of graphed data normalized to the maximum signal from three different experiments is shown in Figure 2b. In this example, we examine translation elongation rates in both non-activated polyclonal T cells and activated OT1 transgenic T cells.
  **Figure 2.**
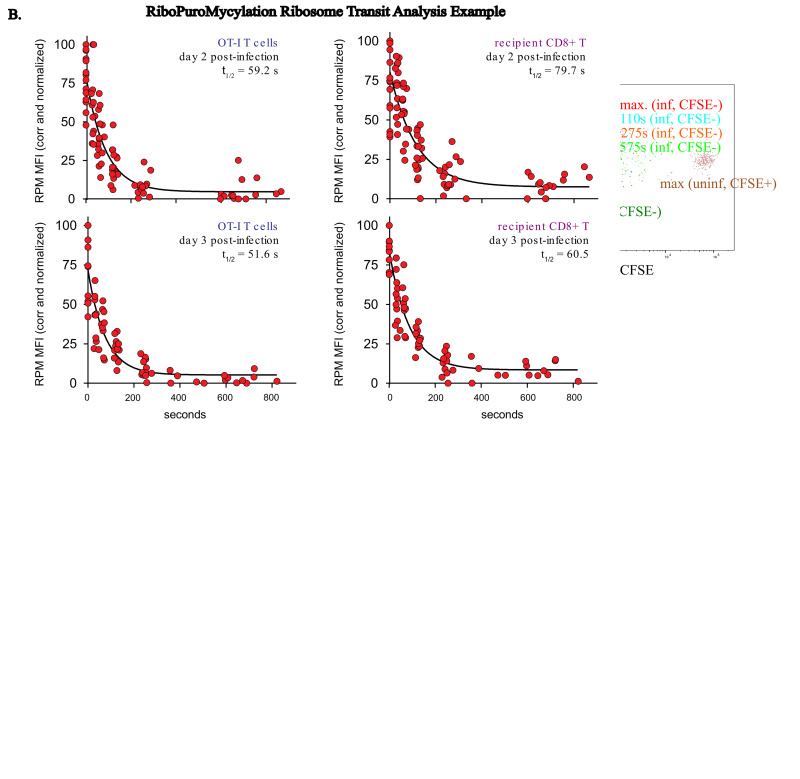
Example gating strategy and RiboPuroMycylation ribosome transit analysis data A. On the left: Gating on OT-1 T CD8^+^ T cells. Singlets by FSCa and FSCw, lymphocytes by SSCa and FSCa, live cells by EMA-, CD3^+^CD19-, CD8^+ ^T cells by CD8^+^CD4-, adoptively transferred cells by CD45.1^+^CD4-, and OT1 T cells by Vb5^+^CD45.2-. On the right: **R**ibo**P**uro**M**ycylation (RPM) staining of divided (CFSE-, infected mice) or undivided (CFSE^+^, uninfected mice) OT-1 CD8^+ ^T cells, with an example of harringtonine runoff after different harringtonine treatment times in CFSE- cells. B. RPM-**R**ibosome **T**ransit **A**nalysis (RTA) of adoptively transferred activated OT-1 T cells or un-activated (resting) host CD8^+^ T cells in mice infected for 2 or 3 days with VACV-SIINFEKL. Three to four independent experiments combined. Normalized by setting the corrected maximum RPM signal (no runoff) to 100.

## Validation of protocol

This protocol or parts of it has been used and validated in the following research article:

- Seedhom et al. [8]. Paradoxical imbalance between activated lymphocyte protein synthesis capacity and rapid division rate. eLife.

## General notes and troubleshooting

1. While extensively validated in lymphocytes and CFSE-labeled lymphocytes from the spleen, thymus, and lymph nodes in two different strains of mice (C57BL/6J and FVB/N) as well as RAG1ko OT1 TCR transgenic mice, this protocol relies on intravenous delivery of protein synthesis modifiers and detection reagents that work similarly in many mammals, so we expect it to be applicable to many other model organisms and most cells in tissue types that are well-vascularized that can be processed to single cells.
2. The reagents puromycin and digitonin vary somewhat from lot to lot and especially from the company purchased. We suggest piloting these reagents ahead of time in comparison experiments, and then ordering and freezing these reagents in bulk for future studies.
